# Predictive Utility of the Functional Movement Screen and Y-Balance Test: Current Evidence and Future Directions

**DOI:** 10.3390/sports13020046

**Published:** 2025-02-08

**Authors:** Adam C. Eckart, Pragya Sharma Ghimire, James Stavitz, Stephen Barry

**Affiliations:** 1 Department of Exercise Science, Kean University, 1000 Morris Avenue, Union, NJ 07083, USA; pghimire@kean.edu (P.S.G.); barryste@kean.edu (S.B.); 2 Department of Athletic Training, Kean University, 1000 Morris Avenue, Union, NJ 07083, USA; jstavitz@kean.edu

**Keywords:** musculoskeletal injuries, functional movement screen, Y-Balance Test, injury risk factors, workload, injury prediction, machine learning

## Abstract

Musculoskeletal injury (MSI) risk screening has gained significant attention in rehabilitation, sports, and fitness due to its ability to predict injuries and guide preventive interventions. This review analyzes the Functional Movement Screen (FMS) and the Y-Balance Test (YBT) landscape. Although these instruments are widely used because of their simplicity and ease of access, their accuracy in predicting injuries is inconsistent. Significant issues include reliance on broad scoring systems, varying contextual relevance, and neglecting individual characteristics such as age, gender, fitness levels, and past injuries. Meta-analyses reveal that the FMS and YBT overall scores often lack clinical relevance, exhibiting significant variability in sensitivity and specificity among different groups. Findings support the effectiveness of multifactorial models that consider modifiable and non-modifiable risk factors such as workload ratios, injury history, and fitness data for better prediction outcomes. Advances in machine learning (ML) and wearable technology, including inertial measurement units (IMUs) and intelligent monitoring systems, show promise by capturing dynamic and personalized high-dimensional data. Such approaches enhance our understanding of how biomechanical, physiological, and contextual injury aspects interact. This review discusses the problems of conventional movement screens, highlights the necessity for workload monitoring and personalized evaluations, and promotes the integration of technology-driven and data-centered techniques. Adopting tailored, multifactorial models could significantly improve injury prediction and prevention across varied populations. Future research should refine these models to enhance their practical use in clinical and field environments.

## 1. Introduction

Injury risk screening has been extensively studied, with the primary goal of accurately predicting first-time injuries. In recent decades, field expedient screens have gained popularity in rehabilitation, sports, and fitness training settings owing to their appeal among practitioners looking to customize programs for their patients and clients. Among the most popular of these screens are the Star Excursion Balance Test (SEBT)/Y-Balance Test (YBT) and the Functional Movement Screen (FMS). Although earlier research demonstrating the predictive value of these screens showed promise, more recent studies have raised several concerns regarding their validity. The most pressing concerns are whether performances in a limited but fundamental set of movements can forecast future performance in a broader range of movements, and whether faulty fundamental movements can predict injury. Early research challenged this idea based on the evidence available at the time. However, acceptable levels of reliability, perceived practical utility, and a lack of studies on validity may have delayed further investigation [1,2]. Since then, several systematic reviews and meta-analyses have attempted to quantify the overall injury-predictive capabilities of movement screens, often recommending caution or suggesting that they should be used alongside other methods. Despite numerous studies in this field, few updated approaches to injury risk assessment have been proposed. In this review, we will address the following questions: What are the theoretical underpinnings of movement screens? Are field-based movement screens effective in predicting injury risk? What factors affect the predictive value of movement screens? Are the underlying premises of current movement screens valid? What other factors affect injury risk? Where do we go from here?

## 2. Materials and Methods

To thoroughly examine the research on this topic, we conducted several literature searches using various databases, including PubMed/MEDLINE, Scopus, Web of Science, ScienceDirect, Cochrane Library, PsycINFO, and SPORTDiscus. We explored different combinations of keywords from the following list: musculoskeletal injury risk factors, Y-Balance Test (YBT), Star Excursion Balance Test (SEBT), Functional Movement Screen (FMS), injury prediction, systematic review, meta-analysis, and injury prevention. For inclusion in the review, we considered investigations such as systematic reviews, with or without meta-analyses, prospective cohort studies, epidemiological studies, cross-sectional studies, and case–control studies pertaining to the predictive validity, sensitivity, positive predictive value, and construct validity of the FMS and YBT. Narrative reviews that provided a critical appraisal of the FMS or YBT were also included to offer contextual perspectives. Study abstracts were screened and categorized based on publication date, relevance to our research questions, and level of evidence. To ensure that relevant studies were not overlooked, we consulted the bibliographies of pertinent studies, such as recent systematic reviews. We prioritized studies published in the last 15 years that demonstrated both high relevance and a strong level of evidence. Since the predictive value of the FMS and YBT has been widely studied, recent meta-analyses that addressed specific themes or research questions were included, while individual studies related to similar themes or research questions were excluded. Studies involving children younger than high school age were excluded. Earlier works discussing the theoretical, historical, and biomechanical foundations of movement screens were included but limited. We excluded studies based on irrelevance, duplication, insufficient focus on validity, or a failure to contribute new information or context in addressing the research questions.

## 3. Results

**Figure 1:**
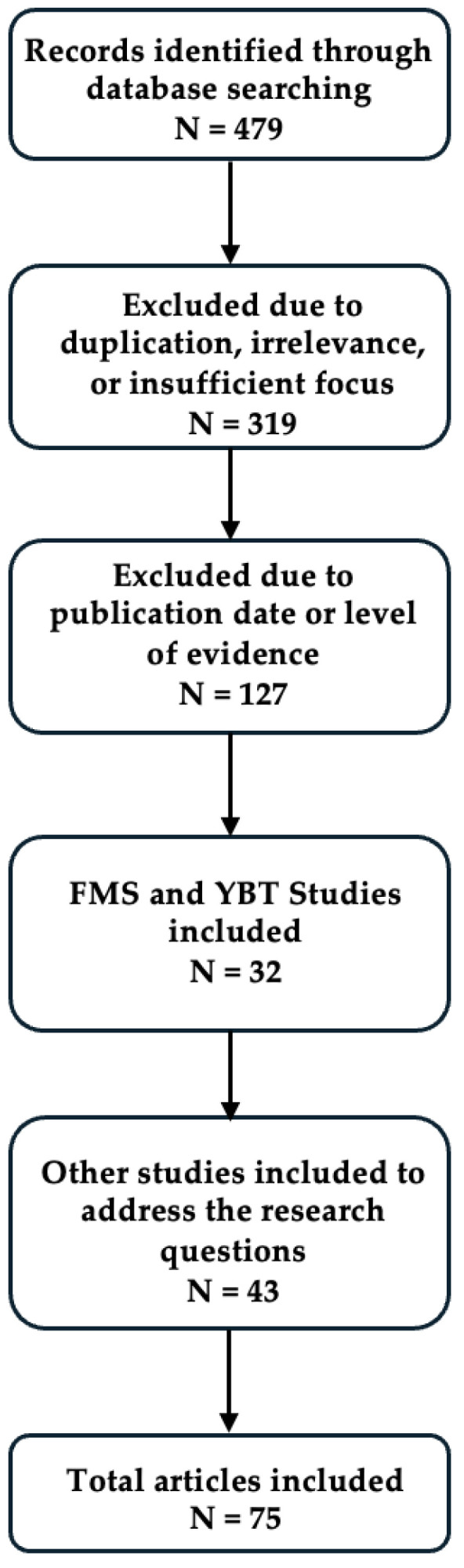
Article curation and exclusion process.

The literature search yielded a total of 479 records, of which 319 were excluded for duplication or for not sufficiently addressing any of the research questions (Figure 1). Additionally, 127 were excluded due to a publication date older than 15 years or because a study with a higher level of evidence, such as a meta-analysis, was available to address a specific theme. After exclusion, 32 studies pertaining specifically to the predictive value of the FMS or YBT remained. The total review included 75 studies, comprising five theoretical framework studies, 22 systematic reviews with meta-analyses, eight systematic reviews, six narrative reviews, 18 prospective cohort studies, 10 cross-sectional studies, four case–control studies, and two animal studies. Table 1 displays the included studies and the key outcomes of each study.

## 4. Discussion

### 4.1. Theoretical Underpinnings

Musculoskeletal injuries usually result from excessive passive loading of tissues due to external forces, delayed or inadequate motor responses to sensory stimuli, or impaired afferent signals caused by disease or previous injury. Extensive research on the relationship among muscle performances, biomechanics, and clinical outcomes has helped shape an accepted theoretical framework on which injury risk assessment approaches are based. Four core tenets can describe this framework:

1. Movement performances depend on the kinetic chain, which encompasses the synergistic behavior of bone, muscles, connective tissues, and nerves spanning multiple joints. Closed-chain exercises involving fixed distal body segments help promote functional joint stability;
2. Muscle performance deficits cause compensatory behavior in local or adjacent body segments, promoting fatigue and decreasing overall movement efficiency. For example, one proposed cause of knee valgus is weak ipsilateral gluteus medius [58];
3. Compensatory patterns are dictated by the forces acting on a joint (e.g., tension, compression, shear, etc.). For example, valgus knee responses lead to anterior tibial shear forces contributing to anterior cruciate ligament (ACL) injury [75];
4. Joint and muscle performances depend on the performance of proximal or distal segments, a phenomenon known as regional interdependence [74,76].

Since muscle synergies among joint agonists, antagonists, and other kinetic chain segments proximal to the primary effectors facilitate human movement, compensatory movement is often viewed as a pervasive manifestation of suboptimal muscle synergies at one or more loci.

Key insights into motor learning and control processes have shaped the understanding of the mechanisms involved in compensatory movement. Early research demonstrating autonomous task-based muscle synergies in response to spontaneous perturbations highlighted the specificity of neural adaptations to environmental stimuli [31,69]. The biomechanical outcome of specific movements is a byproduct of neuromechanical tuning, which relies on task-specific sensory inputs that, in turn, refine the central program generator (CPG) involved in the task. Furthermore, the variability in the CPG pattern for a given task is history-dependent, emphasizing the plasticity of neuromotor activity and underscoring the importance of movement experience for future performances. Although the exact mechanisms governing neuromotor control remain unclear, experiments on the effects of attentional foci on muscle activity have provided practical applications for sports training and rehabilitation. While the internal focus of attention (IFOA) involves focusing on the specific actions of muscles and limbs during a movement, the external focus of attention (EFOA) involves conscious selective attention directed towards the outcome, creating task-specific constraints on coordination. EFOA has been convincingly shown to enhance motor efficiency for functional skills compared to IFOA [77]. Since biomechanics are influenced by local muscular imbalances and task-specific neuromuscular deficiencies, this supports the idea that biomechanical limitations are better assessed in situ—within the context of a functional movement rather than in isolation.

Growing evidence indicates that training and rehabilitation programs focused on functional context yield comparable, if not superior, outcomes to interventions aimed at local muscles or joints [74]. As a result, there has been a significant rise in the popularity of task-based programs within the rehabilitation community despite their relatively slow adoption [76]. In contrast, extensive research extolling the advantages of ground-based strength and power training for athletic performance prompted the swift adoption of kinetic chain approaches in the fitness and sports performance sectors. When movement screens like the FMS and YBT became commercially accessible, reliable methods for assessing functional limitations from a biomechanical standpoint were absent for non-clinical professionals, such as personal trainers or strength and conditioning coaches [15]. Consequently, the FMS (later bundled with the YBT) gained considerable popularity. It provided exercise professionals with a perceived competitive edge by offering a quasi-clinical assessment to optimize exercise prescriptions, address injury prevention, and improve overall training effects. However, in the 20-plus years since the introduction of the SEBT/YBT and FMS, mounting research has cast doubt on the capacity of criterion-referenced movement screens to predict injuries by identifying movement compensations.

### 4.2. Current Evidence on the Predictive Value of Field-Expedient Movement Screens

#### 4.2.1. Star Excursion Balance Test/Y-Balance Test

The YBT (and its earlier version, the SEBT) emerged from the neuromuscular revolution in training and rehabilitation. The YBT is a dynamic balance test that requires the performer to reach in various directions with one leg while balancing on the other. Reach distances are summed and normalized to lower leg length. Several systematic reviews and meta-analyses have been conducted on the predictive value of the SEBT/YBT [29,56]. Gribble et al. conducted an early systematic review of the validity of the SEBT to predict lower extremity injury risk [29]. Only one study by Plisky et al. on the predictive value of the SEBT was included in the analysis due to high heterogeneity among the studies meeting the inclusion criteria [78]. In that study, basketball players with anterior reach asymmetries of greater than 4 cm were 2.5 times more likely to sustain lower extremity injuries. Furthermore, girls with a composite reach score below 94% of their limb length were 6.5 times more likely to experience a lower extremity injury. Recently, Plisky and colleagues conducted an updated systematic review and meta-analysis on the predictive validity of the YBT-LQ (lower quarter) [56]. The inclusion criteria were narrowed to studies in which the YBT assessment kit or YBT procedures were strictly followed. Sixteen studies on predictive validity were included. Similar to Gribble et al., a meta-analysis could not be performed due to the high heterogeneity among the included studies. Studies examining the relationship between future injury and reach asymmetries were mixed across various populations, cutoff scores, and injury definitions. In one of the studies included in the review, injuries and surgeries were associated with lower scores in the FMS, but YBT scores were not impacted [10]. Significant differences in reach scores were observed across multiple subgroups, including sex and sport, but no differences emerged when analyzed by competition level. Due to the low number of female studies, they were excluded from the subgroup analysis. Of the 13 included studies, one reported an odds ratio of 3.5 for future injury using a composite score cutoff of 89.6%, with 100% sensitivity and 71.7% specificity. However, other studies utilized a range of composite score asymmetry thresholds and found no significant relationship with future injury. Smith et al. reported a two-fold increase in injury risk using a greater than 4 cm asymmetry cutoff, with a specificity of 72% (moderate) and sensitivity of 58% (poor) [62]. Lai et al. also reported poor sensitivity and specificity when cutoff scores were optimized through receiver operating characteristic (ROC) analysis [39].

#### 4.2.2. The Functional Movement Screen

The FMS is a criterion-based screening tool for assessing mobility and stability deficits, left–right asymmetries, and pain, evaluated through performances in seven so-called fundamental movement patterns [15]. The creators assert that FMS tests encompass both simple and complex movement patterns, with performances in these movements being interdependent. Scores from all subtests are compiled to create a composite score intended to quantify overall motor competence. Early studies confirmed that a cut score of 14 or lower was linked to a significant increase in injury risk [35]. Nevertheless, these findings faced criticism due to their poor study design and insufficient support for the unidimensional construct of the FMS [1]. A factor analysis study involving marine officer candidates and another involving elite athletes discovered weak factor loadings in FMS subtests, resulting in incoherent factor structures [34,41]. Conversely, a study of a general healthcare sample with 1113 participants revealed a two-factor structure categorized as “basic” (FMS tests: shoulder mobility and active straight leg raise) and “complex” (FMS tests: deep squat, hurdle step, in-line lunge, trunk stability push-up, and rotary stability) movements [37]. Overall, however, component coefficients ranged from weak to moderate, and the internal consistency of the FMS scoring fell below acceptable levels for all but one model [34,37,41].

In a meta-analysis involving six studies with active adult samples (including athletes, military personnel, and firefighters), the authors reported an overall sensitivity of 24.7%, a true positive value of 42.8%, and a true negative value of 72.5% [17]. In another meta-analysis that examined nine studies on athletes and military personnel, an FMS score of 14 or lower was linked to a pooled odds ratio of 2.74 for sustaining an injury [8]. However, the authors acknowledged several limitations, such as differences in the optimal cutoff score for males and females, discrepancies in the definition of injury, and methodological challenges in assessing individual subtest score variance. Additionally, three studies involving 225 participants did not find a statistically significant correlation between FMS scores and injury risk.

Moore et al. conducted a meta-analysis of 29 prospective studies to clarify research gaps and identify factors influencing the relationship between FMS scores and injury risk [48]. Notable findings from the pooled analysis of 36 studies indicate that for FMS scores of 14 or lower, the odds of injury tended to be higher in sports with a greater prevalence of contact injuries, but were inconsistent. The odds were significantly higher for injuries defined by limited or full training or match time loss, and were greater in males compared to females. The specificity and sensitivity of scores of 14 or lower to identify increased injury risk varied widely among all subgroups. However, specificity was found to be higher than sensitivity overall. Furthermore, having one or more asymmetries significantly increased the odds of all-cause injuries when defined by limited or full training or match time loss, and resulted in higher sensitivity for injury risk, regardless of age or injury type. Pain experienced during one or more subtests was significantly associated with increased odds of injury in senior athletes, but not in junior athletes. Nonetheless, all three studies concluded that effect sizes for the relationships between the FMS scores and injury risk were likely not clinically meaningful.

### 4.3. Factors Affecting the Predictive Validity of Movement Screens

Several studies have either advised against using movement screens or suggested their use alongside other factors [6,8,17,48,49]. Some early reviews failed to assess confounding variables such as sex, sports, or injury definitions, resulting in conflicting findings [8,17]. Subsequently, Moran et al. reported that there were only a few FMS studies within military and athlete cohorts that had sufficient homogeneity to conduct a meta-analysis. They concluded that the actual effect size was small [49]. In a more recent review, Moore et al. confirmed Moran’s findings, noting that variations in athlete age, sex, sport type, and asymmetries accounted for some of the mixed results in FMS injury risk studies. Overall, review studies on the FMS and YBT indicated significant variations in composite scores and asymmetries across sport, sex, and age and recommended further research to establish the unique cutoffs that correlate with good predictive validity in each subpopulation [48,49,56].

#### 4.3.1. Age and Sex

In the general population, age strongly predicts musculoskeletal disorders, such as knee osteoarthritis and lower back pain [22]. However, since age closely correlates with numerous other risk factors, analyses often employ age-matched controls or narrow age ranges to provide more pertinent estimates for particular age groups. For example, after adjusting for age, Eckart et al. reported diminished associations between a multifactorial risk model and injury rates in a sample representative of the US population [22]. In a meta-analysis examining risk factors for knee osteoarthritis, age was significant, although obesity and prior injury contributed the most to population-attributable risk [60]. In another meta-analysis, the prevalence of low back pain was generally higher in females compared to males, with prevalence in females varying across age ranges from 6 to over 50 years. However, sex differences in low back pain were not evident in studies with narrow age ranges [79]. A meta-analysis focused on military personnel indicated that, while age was not a significant predictor in studies with a narrow age range, substantial differences were identified when the age range was expanded [57]. This implies that other predictors, such as sex, BMI, physical activity levels, as well as occupational factors, may serve as more reliable injury predictors when samples are classified by age.

Sex influences vulnerability to certain injuries. For instance, females face a higher risk of ACL injuries, whereas males are more susceptible to shoulder injuries related to instability [47]. Overall evidence suggests that females achieve higher composite FMS scores and perform better in flexibility and balance movements, aligning with known sex-based musculoskeletal traits [6,10,54]. Several studies assessing the predictive validity of the FMS and YBT in athletic populations reveal sex differences in both composite and component scores while showing no significant difference in injury risk [5,36,54]. However, Kapnik et al. discovered that cut-off scores maximizing sensitivity and specificity were lower for males (≤11) than for females (≤14). Nevertheless, sensitivity and specificity were considerably higher for females (60% sensitivity, 61% specificity) than for males (22% sensitivity, 87% specificity) [36]. In a factor analysis study, Gnacinski et al. noted a lack of measurement invariance within the sexes, indicating that the suggested ≤14 cutoff score did not apply equally to both sexes and implying that distinct cutoff values would be necessary for male and female groups [28].

#### 4.3.2. Injury Definitions

As previously noted, several systematic reviews have raised concerns regarding the inconsistencies in the definitions and mechanisms of injury, complicating the execution of meta-analyses [48,49]. Nonetheless, Moore et al. found no significant correlation between injury definitions and FMS scores, although sensitivity was highest for injuries characterized by match time loss [48]. The FMS composite score showed slightly higher sensitivity to non-contact injuries compared to all-cause injuries, though it was based on only a few studies focused on non-contact injuries. Moore et al. recommended that future research adopt a more precise definition of injury, including only serious injuries associated with significant time loss (e.g., one week or more) [48].

#### 4.3.3. Injury History

It is well established that previous injuries significantly increase the risk of recurrent injuries, and growing evidence suggests that a history of injuries raises the risk of new injuries in different areas [27,70]. For instance, studies have demonstrated a link between ACL, groin, and back injuries and the occurrence of subsequent hamstring injuries [70]. Although it may be an oversimplification, reasonable explanations for the heightened risk following an initial injury include changes in biomechanics and motor control, as well as a return to excessively high training and competition loads after recovery. However, a reliable biomechanical model that prospectively predicts injury, independent of injury history, has yet to be developed. Nonetheless, many studies on injury risk exhibit methodological flaws by attempting to identify risk factors through retrospective designs [13]. Since injury risk screens are intended to predict future and first-time injuries, analyzing scores after injury undermines their overall utility. Some research indicates that the FMS identifies pre-existing injuries rather than detecting predisposing risk factors [47,70]. However, one study revealed that the FMS composite score could not identify prior ACL injuries in a cohort of female collegiate athletes due to a lack of norm-referenced criterion validity [11].

### 4.4. Are the Premises of Movement Screens Flawed?

The core concept behind an injury-predictive movement screen is its capacity to pinpoint motor control limitations in movement patterns deemed universal to physical functioning. Consequently, only a limited number of idealized movements that supposedly represent essential motor skills are included. Performances in these movements are evaluated against a predefined ideal that is presumed to accurately represent optimal neuromuscular control.

**Figure 2:**
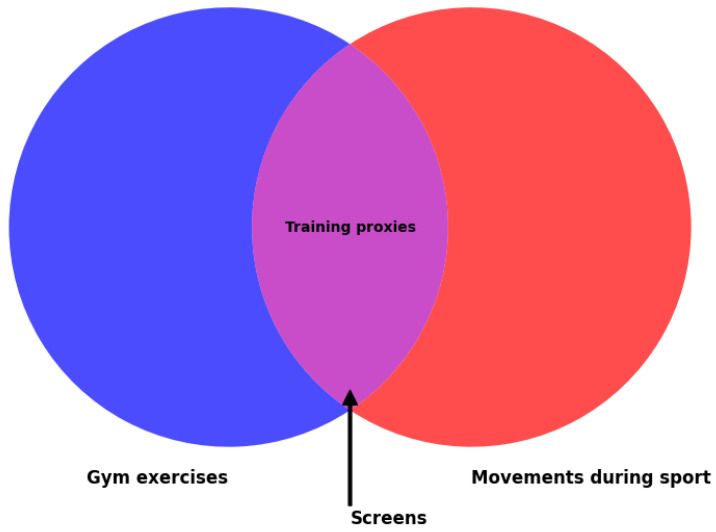
Conceptual framework of movement screening tests.

This system is said to cast a “wider net” to capture functional limitations [15]. For example, if a person has trunk instability, it may manifest as compensatory movements in several FMS movement tests. Similarly, if a person experiences ankle stiffness, it could result in relatively short reaches in the YBT or low scores in the deep squat or in-line lunge. There is limited evidence supporting the validity of this theory. Nevertheless, performance-based movement screens are widely adopted, potentially due to a common logical fallacy. This fallacy assumes that a motor skill, which integrates various general physical characteristics, will effectively assess athletic or functional performance, regardless of the specificity of the movement context. To enhance sport-specific performance, trainers and therapists often employ exercises as proxies for sporting movements (Figure 2). These proxies are further abstracted to represent ‘fundamental’ movements, evolving into ‘movement assessments’ because they are perceived as composites of the essential motor skills necessary for athleticism. This reasoning, illustrated below, is frequently invoked to justify the relevance of movement screens to functional or sporting activities. For instance, dynamic single-leg balance is valued in many sporting contexts due to its face validity for numerous sports movements and its requirement for whole-body motor control, strength, and flexibility. As a result, assessments of single-leg dynamic balance are used to identify functional limitations and serve as a foundation for exercise programming. To illustrate the underlying logic, let C represent the plant phase of the lower body during a baseball pitch; B denotes the common lunge exercise often performed in training; and A signifies the FMS in-line lunge or the lower limb reaches in the YBT.

If A→B→C, then A→C

While the YBT or in-line lunge can be compared to various sports actions, several critical factors regarding its applicability are frequently overlooked. These include the experience of performing the test and the differences in fatigue effects, forces, velocities, kinematics, and unique coordination patterns manifested in the test compared to similar actions during gameplay.

Another example pertains to the deep squat in the FMS. The FMS deep squat is an overhead squat performed with a dowel and is sometimes utilized as a stand-alone test of athletic gross motor function due to its high demands on neuromuscular coordination, trunk stability, and joint mobility. Its direct connection to athletic performances appears restricted to specific sports like Olympic weightlifting or CrossFit. Nevertheless, the rationale for employing the overhead squat assessment is frequently rooted in the assumption that proficiency in this proposed proxy of whole-body motor control implies general athletic ability. One potential reason for the widespread acceptance of this assumption is the common mistake of attributing causes when only associations exist. Athletes are exposed to various training regimens and are more likely to encounter exercises similar to those in movement screens. It is also plausible that athletes who engage in demanding exercises like the deep squat are more inclined to try new exercises, resulting in a broader movement repertoire.

Indeed, athletes engage in a wider variety of exercises and movements than their sedentary counterparts. However, the question arises of whether criterion-referenced proficiency in these exercises provides resilience against injury. If that were true, evidence would indicate a relationship between higher screen scores and athletic event performances. Nonetheless, a systematic review of the association between the FMS and athletic performance challenges the validity of movement performance assessments by proxy [53]. In one study, the deep squat identified only minor deficits in athletic field test performances [53]. In other studies, the deep squat, along with other gross motor patterns, such as the in-line lunge and hurdle step, did not predict athletic performance [53]. Also important is the evidence showing a practice effect on subsequent FMS scores that is not explained by the scoring criteria. Frost et al. asked a group of firefighters to perform the FMS without prior knowledge of the scoring criteria [26]. After the initial trial, firefighters were informed of the criteria for each score, resulting in improvements in composite scores in the second trial. This suggests that scores partially reflect performance expectations rather than genuine motor deficiencies.

With the noted bias toward experience, the next question is whether the FMS scoring criteria are sensitive enough to detect compensations related to injury. The initial screening procedures require measurements of key body segments used to score specific tests. For instance, the shin length measured from the floor to the tibial tuberosity is used to set the height of the hurdle step and position the feet in the in-line lunge. For the deep squat, if the criteria for a score of 3, considered optimal performance, are not achieved after several attempts, the approximately 1.5-inch-thick board that comes with the FMS kit is placed under the heels. This effectively “lengthens” the posterior chain and “increases” ankle dorsiflexion by allowing more degrees of freedom between two fixed points—the feet and the dowel held overhead with both hands. Curiously, the height of the heel raise is entirely arbitrary and universal to all participants, regardless of individual morphologies. This modification might lead to successful completion, corresponding to a score of 2, considered “satisfactory”. This presents yet another conundrum. Although the score decreases by one, the compensation is not necessarily flagged as problematic, suggesting that the criteria for a score of 3 might be an unreasonable standard. A more glaring example of an unreasonable standard is the criteria to score a 3 on the rotary stability test. In the quadruped position, with the hands and knees kept approximately 4 inches apart (the width of the FMS board), participants must perform an ipsilateral arm/leg reach without losing balance. If three attempts to perform this are unsuccessful, the participant is asked to perform the same action contralaterally, which offers significantly more stability. Once again, if successful, the performance is scored at 2, which is deemed satisfactory. These examples highlight the inconsistency in the FMS scoring system by acknowledging participants’ tendency to compensate strategically while incorporating arbitrary modifications as a response to an unreasonable standard.

Movement screens may only be predictive to the extent that a specific test involves joints or body segments that are integral to a participant’s sport or function, or when the scoring criteria reveal injury-specific deficiencies. For example, Chorba et al. analyzed the relationship between FMS scores and lower extremity injuries in a female cohort from various sports [11]. The association between FMS scores and injury was stronger when shoulder mobility scores were excluded from the total score. The composite score includes scores from upper and lower body subtests, which undermines the overall predictive sensitivity for athletes whose injuries primarily involve the areas used during sport, such as the lower extremities in soccer players. However, evidence suggests that the validity of the subtest criteria is also questionable. The scoring criteria were developed based on the assumption that specific compensations are inherent in fundamental movement patterns, and that the differences in scores would indicate specific levels of dysfunction. Recent studies have challenged this assumption, demonstrating considerable overlap in joint ranges of motion (ROM) across FMS subtest scores [4,30]. In one study, ankle mobility restrictions were indicated by lower scores in the deep squat and in-line lunge. However, lower scores did not always predict ankle mobility limitations [30]. Moreover, there was significant variability in the ROM of the ankle, hip, and shoulder associated with FMS scores. In a similar study, shoulder ROM during the deep squat differed only between scores of 1 and 2. Recent findings also suggest that generalized joint laxity was associated with higher DS scores, indicating that genetic variations in joint structure may explain scores independently of dysfunction or injury [80].

A balanced critique of movement screens acknowledges some evidence of predictive validity and seeks to examine the apparent links. When viewed through the lens of specificity, it becomes clearer why the FMS and YBT may sometimes predict injury. As stated earlier, fitter individuals who engage in a variety of exercises possess greater strength, stability, and mobility, which serve to confer injury protection and might explain the higher FMS scores [37]. Moreover, since these screens mimic common exercises performed in the gym, criterion bias tends to favor those with diverse exercise experience [26]. However, the problem is that these screens generalize functional limitations observed in specific movements to all movements without taking into account their specificity to the movement goal. Consequently, the evidence regarding causal links between higher FMS scores, greater fitness levels, and decreased injury rates is inconsistent. For example, Tehyan et al. observed reduced injury rates among those with higher FMS scores and better fitness test scores in military cohorts, whereas Svensson et al. found no correlation among athletic performance tests, FMS scores, and injury rates in football players [65,66,67]. Many studies show improvements in FMS scores and injury rates following various interventions, regardless of the specific characteristics of each cohort [1]. However, it remains unclear whether experience in performing the FMS tests or specific training interventions led to improved FMS scores due to a lack of studies controlling for the practice effect.

### 4.5. Other Injury Factors

#### 4.5.1. Occupational Risk Factors

Occupational characteristics expose individuals to significant risk factors for injury, and should be included in injury risk assessments. Several broad categories of risk for musculoskeletal injuries (MSIs) and other occupational musculoskeletal disorders have been identified, including shift work, repetitive movements or positions, high physical exertion, prolonged sedentary work, computer use, and sleep-related issues.

Similar to the exercise training paradigm, the relative workloads of physical activity influence the rates of MSIs in occupational cohorts. However, being inactive at work can be equally harmful, if not more so. While cross-sectional studies indicate that sitting for long periods at work significantly raises the risk of low back, neck, and shoulder pain, standing for more than four hours a day in occupational settings has also been linked to an increased risk of low back symptoms [14,20]. Additional evidence points to a meaningful relationship between occupational loading and spinal disc degeneration [45].

In a random sample of 3710 French workers aged 20 to 59, high physical exertion and working with arms above shoulder level accounted for 30% and 7% of all upper-extremity injuries, respectively [50]. Supporting these findings, a large-scale epidemiological study by the World Health Organization (WHO) and the International Labour Organization (ILO) reported that force exertion, demanding postures, repetitiveness, hand-arm vibrations, lifting, kneeling, squatting, or climbing for 2 or more hours a day significantly increased odds ratios for osteoarthritis of the knee or hip compared to low or no exposure [32].

In addition to intraday work demands, shift work is linked to inadequate recovery between workdays. A recent meta-analysis of 29 high-quality studies found that the risk of occupational accidents was significantly increased by night shifts compared to morning shifts, the number of consecutive shifts, work shifts exceeding nine hours, and reduced work breaks [25]. The risks tied to shift work affect all shift-based occupations, but they are particularly evident in allied health professions, which involve long shifts, prolonged standing, and repetitive patterns and postures [12,19,23,43]. Multiple meta-analyses involving healthcare providers demonstrate significant associations between postures, patient volume, work time loss, degenerative spine disease, and pain in the lower back, neck, and shoulders [12,19,23,43]. These associations are explained by fatigue, worsened by a gap between work demands and physical preparedness which plays a crucial role in the risk of MSIs and should be factored into injury screenings for the general population.

#### 4.5.2. Joint Laxity

Joint laxity, or hypermobility, is characterized by excessive ranges of motion and could be caused by a heritable phenotype, injury, or an adaptation to repeat exposures. The Beighton score (BS) is a field-expedient generalized joint laxity screen with good reliability and injury-predictive value [38,42,52]. Although it is not typically used in general fitness settings, its ease of use and low technical requirements make broader adoption among non-clinical exercise professionals more feasible.

However, the optimal cutoff values for the BS concerning age, sex, and ethnicity have only recently been established. Singh et al. evaluated Generalized Joint Hypermobility (GJH) using the BS scoring system in 1000 males and females aged 3–101. Generally, females and non-Caucasians had higher BS scores across the lifespan. A cut-off score of ≥4 demonstrated 80% sensitivity and 99.3% specificity for females aged 40–59 and males aged 8–39. However, using the ≥4 cutoff for both sexes across the lifespan resulted in a 60% false-positive rate.

Malek et al. note that the BS’s ability to accurately reflect GJH remains controversial, as the joints represented in the scoring system are primarily the upper limbs, overlooking several major joints, thereby hindering a direct identification of GJH [46]. The researchers concluded that the BS should not be employed as the main tool for differentiating between localized and generalized hypermobility, nor should it be used in isolation to rule out the presence of GJH. Supporting this perspective, several studies have reported no connection between GJH and an increased risk of musculoskeletal injuries [46]. Nevertheless, a significant methodological distinction between conflicting studies lies in the inclusion of injury rates per unit of exposure, which may account for differences in outcomes.

Interestingly, Armstrong (2020) discovered a weak yet significant relationship between BS and the FMS composite score in college-aged female dancers. Additionally, some weak but significant associations were found between total BS and individual components of the FMS or SEBT. No relationships existed between the FMS and SEBT composite scores, or between the SEBT composite score and total BS [80].

#### 4.5.3. Sleep

Evidence shows that sleep disruptions negatively affect both physical and mental performance, hinder recovery from physical exertion, and are linked to injuries in occupational, athletic, and tactical populations [9]. A meta-analysis with 268,332 participants reported a 62% increase in the risk of all-cause work-related injuries, and attributed 13% of these injuries to sleep issues [71]. Another recent meta-analysis focusing on military cohorts identified significant effects of sleep disturbances on injury risk while controlling for other strong predictors [44]. Ruan et al. discovered that sleep quality, as measured by a Pittsburgh Sleep Quality Index (PSQI) score of less than 7, serves as an independent risk factor for MSI in basic training recruits [59]. The relationship between sleep quality and MSI risk is likely linked to disruptions in circadian rhythms and the dysregulation of the hypothalamic–pituitary–adrenal (HPA) axis [9]. In individuals with chronic pain, sleep quality predicts next-day pain, and vice versa [3]. Paradoxically, poor sleep quality may signal overtraining syndrome, or could be a contributing factor, highlighting the importance of effective workload-to-recovery management in at-risk populations [9]. Therefore, sleep quality is a critical risk factor to consider when evaluating injury risk.

#### 4.5.4. Multifactorial Injury Risk Models

Bahr (2016) noted that the populations in which movement screens are administered tend to be relatively homogeneous from a performance standpoint, leading to substantial overlap in scores among individuals at different risk levels within each sample, which may result in prediction errors [7]. This is evidenced by weak relationships and confounding factors when risk ratios are aggregated. Additionally, Bahr emphasized the importance of incorporating both non-modifiable (e.g., age, sex, injury history) and modifiable risk factors (e.g., strength, stability, mobility) to enhance predictive accuracy [7]. However, few injury risk studies exist that integrate movement screens with other modifiable and non-modifiable risk factors. An early study by Lehr et al. included 183 collegiate athletes across ten sports, utilizing aggregated movement screen scores [40]. Evidence-based cut-off points specific to competition level, sport, injury history, and gender were employed to establish low- and high-risk categories. Those categorized as high risk were over three times as likely to sustain a non-contact lower extremity injury. While the findings are promising, limitations such as a small sample size from a single location and a lack of test reliability reporting call for confirmatory studies. Rhon et al. conducted a meta-analysis involving military personnel, identifying significant injury risk factors such as female sex, high BMI, pain during FMS tests or a score of ≤14, and poor fitness test scores [57].

Similarly, Teyhen and colleagues reported a 90% test sensitivity for high injury risk when military personnel presented with three or more of the following self-reported risk factors: smoking, prior surgery, recurrent musculoskeletal injuries, limited-duty days in the previous year due to musculoskeletal injury, asymmetrical ankle dorsiflexion, pain during FMS clearing tests, and poor performance on the 2-mile run and 2-min sit-up test [67]. In another study by Teyhen et al., multifactorial predictors formed a highly sensitive model for time-loss injuries among 922 army soldiers [68].

Other modifiable factors, such as body composition and fitness level, have also been shown to influence the risk of extremity injuries [18,64]. In several studies, low FMS scores were linked to higher BMI and reduced fitness levels [51,73]. Generally, higher BMI, along with other factors like generalized joint laxity, genetic predispositions, injury history, and lower body and core weakness, are associated with ACL injuries across diverse cohorts [16,24,55].

#### 4.5.5. Workloads and Injury

MSI risk is considered to be proportional to the difference between acute and chronic workloads through fatigue mechanisms. Several prospective studies have demonstrated a negative impact on injury risk factors such as postural control, joint position sense, and lower limb strength when acute fatigue is induced [72]. In the meta-analysis by Eckard et al., several notable relationships between load and injury risk were identified [21]. The workload measures in these studies included internal training loads (ITL), external training loads (ETL), and absolute and relative training loads. Common ETLs encompassed session frequency, distance, duration, and repetitions, while ITL measures included questionnaires and session ratings of perceived exertion (sRPE). Recent studies report moderate-strength evidence linking acute-to-chronic workload ratios (ACWRs) with injuries in both athlete and tactical populations [21]. Researchers typically define ACWR as the ratio of the mean training load in the current period to the mean in the previous period. Several ACWRs associated with an increased injury risk have been documented, including daily and weekly ratios. Eckard et al. reported that direct relationships between workloads and injury rates were primarily found in studies utilizing acute absolute loads. Conversely, inverse relationships were mainly observed in studies evaluating chronic loads [21]. This supports the notion that high chronic loads can offer protection against injuries. Research employing a mix of workload measures reveals a U-shaped relationship, where low loads do not trigger protective physiological adaptations, while excessive loads lead to increased fatigue and damage, thus diminishing injury resistance [21]. The frequency of training sessions was an inconsistent external workload measure with no clear link to injury risk; however, the sRPE emerged as the most useful internal workload measure. Ultimately, minor to moderate adjustments in relative workloads correlated with a lower injury risk compared to either very small or very large changes.

Although injury risk assessment based on workloads is promising, this area of research faces similar limitations as movement screens. Presumably, injury rates vary across predisposing factors such as sport, sex, or injury history. However, studies measuring workloads have not differentiated risk for these specific injury factors, which could provide greater predictive accuracy for injuries [21]. Some studies indicate a latency period between workload exposures and increased injury rates, possibly due to the decline in adaptive reserves, which refers to how well an athlete’s HPA axis regulates stress responses and adapts physiological processes to changing demands. Although ACWRs account for differences in physiological stress between periods, they do not consider the adaptive reserve or recovery efficiency between training or sporting bouts. Latency periods may clarify the temporal relationship between workload exposures and injury events. Still, this does not address the challenge of predicting specific injuries, which would require the continuous measurement of local tissue workloads during sports and training activities, as well as monitoring overall on- and off-field workloads, sleep quality, and other recovery determinants.

### 4.6. Emerging Approaches in Injury Prediction

Arguably, basic linear methods for injury prediction have yet to yield models with strong sensitivity or specificity [63]. Some researchers describe injury prediction as a complex system comparable to forecasting hurricane paths [63,81]. Complex behaviors demonstrate nonlinear characteristics, where the effects of interconnected components develop in relation to one another and the outcome. The biomechanical outcome of a specific movement can be understood as an emergent property stemming from the self-organization of a dynamical system that adjusts feedforward and feedback mechanisms using current and past inputs. Following an injury or as a consequence of fatigue, biomechanical components undergo fundamental changes that alter the dynamical landscape and establish a new pattern of movement synergies, which cannot be anticipated through linear associations between components from before the exposure.

**Figure 3:**
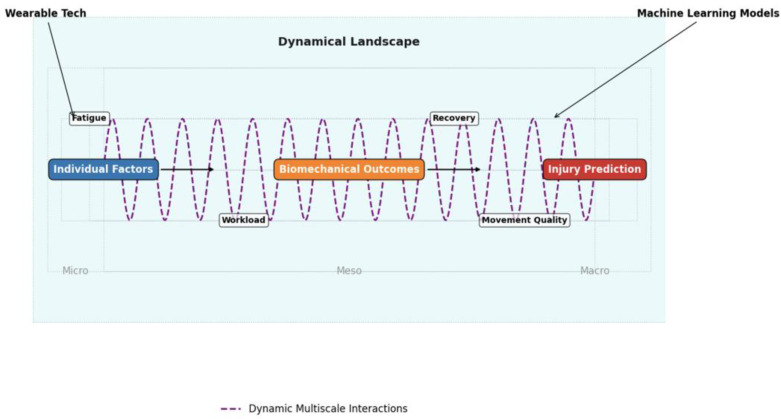
A dynamic and multiscale framework for injury prediction.

However, the core issue affecting biomechanical approaches to injury risk stratification is not merely compensation due to fatigue, but rather capturing the dynamic multiscale interactions of injury factors at the individual level (Figure 3). This necessitates analyses of specific movements and their associated biomechanics, including the precise kinematics, forces, and velocities at which injuries might occur, while considering factors such as gender, age, sport, fitness level, acute and chronic workloads, previous injuries, and indicators of athlete readiness, such as exertional indices (e.g., heart rate, heart rate variability, RPE) and measures of strength and power. To achieve this, wearable or easily deployable technologies are essential for data collection, management, and real-time analysis to develop personalized neuromuscular models.

Machine learning approaches possess the ability to manage complex, nonlinear, multiscale, and dynamic interactions, positioning injury prediction with high-dimensional data as a promising objective. Consequently, studies utilizing nonlinear and machine learning (ML) models have started to arise [82]. Furthermore, the advancement of wireless intelligent monitoring technologies, including markerless motion capture and inertial measurement unit (IMU) sensors that integrate GPS, gyroscope, and accelerometer technology, makes non-invasive methods for modeling and assessing ground reaction forces and tissue loading easily accessible to sports performance coaches, fitness professionals, and rehabilitation clinicians.

Although a comprehensive examination is beyond the scope of this review, it is worth noting that various machine learning methods have been used to investigate injury predictors in different populations, such as athletes, occupational workers, and military personnel, with relatively high accuracy [83,84,85]. These studies often include sensor data, questionnaires, fitness tests, motor skill evaluations, and anthropometric data. However, only a few studies utilizing ML methods have used a combination of multifactorial predictors to assess injury risk prospectively. One remarkable study by Rossi et al. used a multifactorial feature set, including anthropometrics, kinematic, metabolic, mechanical load, and fatigue data collected from GPS/IMU monitoring systems, to prospectively predict injury in professional male soccer players. The decision tree injury classifier achieved a recall of 80% and a precision of 50%, and its predictive accuracy improved as more data were collected throughout the season [84]. Interestingly, previous injuries emerged as a significant predictor when combined with cut-offs for specific workload metrics. To summarize, a previous injury plus a lower value for high-speed running distance in a training session accounted for 42% of new injuries. A previous injury plus a higher value for high-speed running distance and a lower value for total distance covered in a training session accounted for 30% of new injuries. Finally, previous injuries plus higher values for high-speed running distance and a total distance covered of over 2.5 times the player’s average accounted for 28% of new injury cases. However, the homogeneity of the sample, the collection of time-series data during training and sport participation periods, including all relevant features, and the collection of relevant sport/activity-specific workload measurements are essential in producing highly accurate models [33,86]. For example, IMU measurements collected from a one-time Cooper test and questionnaire data produced low-performing ML models in a college-aged cohort of men and women [86]. However, the performances of gender-specific models were better than those of mixed-gender models. In another mixed sample of male and female floorball and basketball players, a combination of biomechanical, joint-laxity, and flexibility tests yielded an AUC ROC of 0.63, which is generally considered poor for diagnostic purposes [33]. Although gender, body composition, and physical features were among the most important, the lack of time-series data that could be used to account for changes incurred by varying workloads throughout training and sports season likely reduced the model’s predictive value.

### 4.7. Practical Considerations

The FMS and YBT show an inconsistent ability to predict injuries. Factors such as sex, age, type of sport, injury history, and physical fitness significantly affect the validity of these screens. Future research should investigate scoring systems for movement screens that consider these differences. While they can be useful in certain situations, risk screens often generalize limitations in contexts where specificity is essential. It is necessary to develop methods of injury risk assessment that focus on specific and crucial sporting actions. Although familiarity with movement tests can enhance scores, it is questionable whether higher scores truly indicate an effective injury prevention capability. Additional research is needed on modifying existing or creating new movement screens that reduce or eliminate prior experience bias. Factors such as repetitive motions, inadequate recovery (e.g., shift work), and sleep disturbances, if considered, may improve the sensitivity of injury risk assessments. Machine learning and wearable devices, like IMUs, offer more accurate and dynamic methods for predicting injury risk through multifactorial models and individualized workload/recovery monitoring. In general, tailored approaches that combine relevant movement/activity assessments with broader risk factors, such as relative workloads, recovery metrics, and past injuries, will improve predictions in injury assessments.

### 4.8. Limitations

Our intention was not to conduct a systematic review. However, higher reporting standards for narrative reviews are essential, as they promote transparency and trustworthiness while providing context for the authors’ conclusions. Therefore, we adopted a structured approach to our literature search and study inclusion methods. We did not adhere to the standard practices for systematic reviews, such as study selection and risk of bias assessment. Consequently, some relevant studies may not have been included in this review. Nonetheless, by incorporating both earlier and more recent systematic reviews and meta-analyses on the FMS and YBT, our perspective reflects the current weight of evidence regarding these screens.

## 5. Conclusions

Field-based screens for musculoskeletal injuries, such as the FMS and the YBT, have limited utility in predicting injuries due to their context-specific nature. Various confounding factors, inconsistent scoring validity, and an overreliance on general movement patterns undermine their effectiveness. Innovative approaches like workload monitoring and machine learning demonstrate greater promise by combining multifaceted data and personalizing risk assessments. Future efforts should focus on integrating movement screens with individualized evaluations that take into account unique exertional, biomechanical, occupational, and lifestyle factors to enhance injury prediction and prevention. This shift toward more dynamic, technology-driven models is essential for increasing accuracy and relevance across different populations.

## Figures and Tables

**Table 1:** Studies included in the review.

Authors	Topic	Study Type	Year	Sample	Key Outcomes
Abeler et al. [3]	Pain and sleep	Prospective cohort	2021	Patients with sleep problems	Pain significantly predicts how well someone sleeps the next night and slightly affects how long they sleep. Sleep quality might also predict pain the next day.
Aleixo et al. [4]	Deep squat and joint mobility	Cross-sectional	2024	College students	DS scores show limited correlations with joint mobility, indicating low convergent validity and discrimination ability.
Armstrong & Greig [5]	Predicting injuries with FMS	Prospective cohort	2018	Rugby players	The FMS composite score significantly predicts total days injured in forwards, despite weak predictive ability.
Asgari et al. [6]	Predicting injuries in females with FMS	Systematic review	2021	Active female adults	The studies exhibited modest quality, with mixed results on FMS predictive validity and suggested higher cutoff values to improve accuracy.
Bahr [7]	Predicting injury with screens	Review	2016	NA	The study identifies challenges in predicting injuries due to overlap in risk levels and a lack of intervention studies.
Beardsley & Conteras [2]	Predicting injuries with FMS	Review	2014	NA	The FMS exhibits strong reliability but its validity is questioned, and low scores do not hinder elite performance.
Bonazza et al. [8]	Predicting injury with FMS	Systematic review, meta-analysis	2017	College sports teams, military personnel	The FMS exhibits intraclass correlation coefficients of 0.81 and scores ≤ 14 correlate with 2.74 times higher injury odds, but validity concerns remain.
Chennaoui et al. [9]	Sleep and injury recovery	Review	2021	NA	This study examines circadian rhythm-related genes, emphasizing their enrichment in muscle development, cellular responses, and metabolism, with implications for myocardial function. Key genes identified include Dicer1, Xbp1, and Srebf1, which may influence memory, immunity, and various physiological processes. However, their specific relationships warrant further exploration.
Chimera et al. [10]	Effect of injury history, sex, and performance on FMS, YBT	Cross-sectional	2015	Division I athletes	FMS composite scores correlate with injuries and surgeries, particularly hip, elbow, and shoulder issues. Performance worsens for knee surgeries and hip injuries, while trunk/back and ankle injuries show better results. Female athletes perform worse in some movement patterns but better in others compared to male athletes, who display greater anterior asymmetry.
Chorba et al. [11]	Predicting injuries with FMS	Prospective cohort	2010	Female, Division II athletes	Eighteen injuries were recorded, mainly lower extremity injuries. A significant association was found between FMS scores of 14 or less and injury occurrence, with 69% of athletes scoring 14 or below sustaining injuries. The odds ratios were 3.85 for all subjects and 4.58 excluding ACLR subjects. Sensitivity and specificity were 0.58 and 0.74, respectively, indicating moderate predictive ability. A strong positive correlation (r = 0.76) was also observed between low FMS scores and injury likelihood.
Clari et al. [12]	Musculoskeletal disorders among perioperative nurses	Systematic review, meta-analysis	2021	Nurses	The highest prevalence of work-related musculoskeletal disorders (WRMSDs) was in the lower back (62%), followed by the knee (47%) and shoulder (44%). Other affected areas included the waist (42%), neck (39%), ankle–feet (35%), upper back (34%), hand–wrist (29%), and elbow (18%). Meta-regression analysis revealed that sex, age, and BMI were not significant predictors of low-back disorders.
Clifton et al. [13]	Challenges in injury prediction	Theoretical framework	2016	NA	The study emphasizes the necessity of incorporating injury incidence data into injury risk factor research. Injury incidence data are crucial for accurately interpreting injury-prediction analyses; without these data, injury-risk estimates may lack accuracy and show increased uncertainty.
Coenen et al. [14]	Occupational exposures and musculoskeletal diseases	Systematic review, meta-analysis	2018	Adults	The meta-analysis indicated that substantial occupational standing (over 4 h per workday) was linked to increased low-back symptoms, with a pooled odds ratio of 1.31. Evidence for lower and upper extremity symptoms was too heterogeneous for meta-analysis; however, most studies reported significant negative associations between occupational standing and lower extremity symptoms, while associations with upper extremity symptoms were inconsistent.
Cook et al. [15]	Fundamental movement screening	Theoretical framework	2006	NA	Research on movement-based assessments is limited, primarily due to the few quantitative tests available. If the FMS can successfully identify these individuals, targeted prevention strategies can be implemented. A proactive, functional training approach can reduce injury risk and enhance performance efficiency, ultimately improving overall wellness and productivity in active populations.
Dauty et al. [16]	Risk factors for ACL injury	Cross-sectional	2022	Athletes	Results indicated that age, BMI, antero-posterior laxity, knee strength, and passive knee alignment were associated with non-contact ACL injuries. Only passive knee valgus was significant for women. The study suggests that hamstring strengthening may prevent non-contact ACL injuries, particularly in younger male athletes or those with knee laxity.
Dorrel et al. [17]	Predicting injury with FMS	Systematic review, meta-analysis	2015	Active adults	The meta-analysis revealed that the FMS has a specificity of 85% but a low sensitivity of 24%, resulting in an AUC of 0.58, indicating slight predictive capability.
dos Santos Bunn et al. [18]	Risk factors for musculoskeletal injuries in military personnel	Systematic review, meta-analysis	2021	Military personnel	Groups at heightened risk for injuries included older individuals, those with overweight or obesity, individuals with previous injuries, and those performing poorly in 1600–3200 m running tests. Factors such as gender, ethnicity, and smoking were not linked to injuries overall. However, subgroup analysis indicated that women had a significantly higher likelihood of developing injuries, with a relative risk of 2.44 in studies with follow-up periods of fewer than 12 months.
Du et al. [19]	Occupational exposures and musculoskeletal diseases	Systematic review, meta-analysis	2021	Nurses	The review highlights the prevalence of these conditions among nurses and identifies key occupational factors that increase risk, such as heavy lifting, repetitive tasks, and high job stress. The findings underscore the significant impact of psychosocial and physical exertion on the musculoskeletal health of nurses, suggesting the need for targeted interventions to reduce these risks and improve occupational health outcomes.
Dzakpasu et al. [20]	Musculoskeletal pain and sedentary behavior	Systematic review, meta-analysis	2021	Adults	A meta-analysis of cross-sectional studies revealed a significant association between full-day sedentary behavior (SB) and low back pain (LBP), with narrative synthesis suggesting potential associations with knee pain, arthritis, and general musculoskeletal pain (MSP). For occupational SB, associations were found with self-reported workplace sitting and LBP, while experimental evidence indicated reductions in LBP, neck/shoulder pain, and general MSP with decreased workplace sitting.
Eckard et al. [21]	Training load and injury	Systematic review	2018	Athlete, military, first responders	47 studies reported statistically significant findings, establishing a clear association between training load and injury risk.
Eckart et al. [22]	Injury risk models in the US population	Case control	2024	Active US citizens	High-risk group membership (age > 40, obesity, lack of muscle-strengthening activities, sedentary lifestyle, low back pain) predicted functional difficulties with 67.4% sensitivity and 87.2% specificity.
Epstein et al. [23]	Musculoskeletal disorders among surgeons and interventionalists	Systematic review, meta-analysis	2018	Surgeons	Pooled prevalence estimates for work-related musculoskeletal disorders include 17% for degenerative cervical spine disease, 18% for rotator cuff pathology, 19% for degenerative lumbar spine disease, and 9% for carpal tunnel syndrome. From 1997 to 2015, degenerative cervical spine disease prevalence increased by 18.3%, while degenerative lumbar spine disease rose by 27%. Pain prevalence among physicians with MSDs ranged from 35% to 60%.
Evans et al. [24]	Risk factors for ACL injury	Prospective cohort	2012	Military personnel	A statistically significant difference in average BMI was found between injured (25.6 kg/m^2^) and uninjured groups (24.4 kg/m^2^). Individuals with a higher-than-average BMI and a narrow femoral notch width were at significant risk for ACL injury.
Fischer et al. [25]	Occupational injuries and work schedule	Systematic review, meta-analysis	2017	Occupational workers	The findings indicate that non-standard work schedules, particularly those involving night shifts and extended hours, are associated with a higher risk of occupational injuries.
Frost et al. [26]	FMS and practice effect	Cross-sectional	2013	Firefighters	The researchers found that when individuals were informed about the grading criteria, their FMS scores improved, suggesting that performance on the screen may be influenced by knowledge rather than solely reflecting underlying movement dysfunctions.
Fulton et al. [27]	Previous injury an injury risk	Systematic review	2014	Active adults	ACL injuries increase the risk of re-injury to the same ACL and to other lower extremity injuries. Hamstring injuries are linked to further injuries in the same hamstring and knee. Individuals with a history of Achilles tendon ruptures are more likely to experience similar injuries on the opposite side. Ankle sprains heighten the risk of re-injury to either ankle.
Gnacinski et al. [28]	FMS factor analysis	Cross-sectional	2016	College athletes	The results indicate support for 1- and 2-factor models, with no significant difference in fit. Measurement invariance was not observed across sexes. Caution is advised regarding sex differences in scores.
Gribble et al. [29]	SEBT and lower extremity injury	Systematic review	2012	Active populations	That the Star Excursion Balance Test (SEBT) is a highly reliable and representative dynamic balance assessment tool, particularly for active individuals. It has proven its reliability, validity, and utility in predicting lower extremity injury risk, identifying dynamic balance deficiencies in patients with various lower extremity conditions, and effectively assessing the impact of training programs on both healthy individuals and those with lower extremity injuries.
Hincapié et al. [30]	FMS and joint range of motion	Cross-sectional	2022	College athletes	Higher FMS task scores were generally associated with greater ROM compared to lower scores across various joints. However, the study also observed a large variation in ROM measurements, indicating substantial overlap in joint ROM among athletes with different FMS task scores.
Horn et al. [31]	Central program generators	Animal study	2004	Intact animals	The study found significant changes in central motor programs and random variability between successive cycles. While some variability seemed random, it was influenced by central history dependency, indicating a complex interplay in the production of functional behavior and potential adaptive strategies in uncertain environments.
Hulshof et al. [32]	Occupational risk factors and osteoarthritis	Systematic review, meta-analysis	2021	Occupational workers	The pooled prevalence of occupational exposure to ergonomic risks was estimated at 0.76, with subgroup analyses revealing variations based on age group, occupation and country, but not by sex.
Jauhiainen et al. [33]	Predicting ACL injuries with machine learning	Case-control	2022	Elite female athletes	Random forest identified 12 consistent injury predictors, while logistic regression identified 20, with 10 overlapping predictors in both models, including sex, BMI, hamstring flexibility, knee joint laxity, medial knee displacement, height, ankle plantar flexion at initial contact, leg press one repetition max, and knee valgus at initial contact. Cross-validated areas under the receiver operating characteristic curve were 0.65 for logistic regression and 0.63 for random forest.
Kazman et al. [34]	FMS factor analysis	Cross-sectional	2014	Marine officer candidates	EFA revealed two components related to separate individual movements (shoulder mobility and deep squat), but the factor structures were not interpretable. Both original scores and scores discounting pain instruction were analyzed, showing similar results and low internal consistency (Cronbach’s alpha of 0.39), indicating a lack of reliability in FMS sum scores.
Kiesel et al. [35]	Predicting injuries with FMS	Prospective cohort	2007	Professional football players	The FMS demonstrated limited sensitivity (0.54), suggesting it might not effectively rule out injury likelihood.
Knapik et al. [36]	Predicting injuries with FMS	Prospective cohort	2015	Coast Guard cadets	FMS scores ≤ 12 were associated with higher injury risk for men, while scores ≤ 15 were linked to higher risk for women. The optimal FMS cut point, based on Youden’s index, was ≤11 for men (22% sensitivity, 87% specificity) and ≤14 for women (60% sensitivity, 61% specificity), indicating moderate prognostic accuracy for injury risk among female cadets but relatively lower accuracy for male cadets.
Koehle et al. [37]	FMS factor analysis	Cross-sectional	2016	Adults	A mean FMS summary score of 13.7 was negatively correlated with age and body mass index. Internal consistency, evaluated using ordinal and Cronbach’s alpha methods, yielded scores of 0.73 and 0.64, respectively. Exploratory and confirmatory factor analyses revealed two main factors: a basic movement factor (including shoulder mobility and active straight leg raise) and a complex movement factor (encompassing squat, hurdle step, inline lunge, and trunk stability push-up). Rotary stability exhibited dual loading but had minimal impact when excluded.
Konopinski et al. [38]	Hypermobility and injuries	Prospective cohort	2012	Soccer players	The study reported an injury incidence of 11.52 injuries per 1000 h and found that 33.3% of players had hypermobility. Hypermobile participants had a significantly higher injury incidence during the season compared to non-hypermobile participants (15.65 injuries per 1000 h). They were also more likely to experience injuries, reinjuries, and severe injuries.
Kraus et al. [1]	Predicting injuries with FMS	Review	2014	NA	The FMS showed reliability with experienced raters, but its total score’s predictive ability for athletic performance was limited, contrasting with its moderate support for predicting injury risk in team sports. Most FMS-based intervention programs improved motor quality, but this lacked confirmation in randomized trials, emphasizing the need for critical expertise in implementing FMS findings practically.
Lai et al. [39]	Predicting injuries with YBT	Case-control	2017	Athletes	The study found no correlation between the laterality of reach asymmetry and the composite score with the laterality of injury. Cutoff scores, including the commonly used 4 cm cutoff from previous studies, demonstrated poor sensitivity and specificity. Additionally, none of the identified asymmetric cutoff scores were associated with an earlier occurrence or increased rate of injury in the multivariate analyses.
Lehr et al. [40]	Predicting injuries with field-expedient screens	Prospective cohort	2013	Athletes	An injury prediction algorithm used to categorize risk for noncontact lower extremity injuries incorporating movement screening performance, demographic information, and injury history was evaluated. Athletes classified as high risk (*n* = 63) had a significantly higher risk of noncontact lower extremity injury (27/63) during the season, with a relative risk of 3.4.
Li et al. [41]	FMS factor analysis	Cross-sectional	2015	Elite athletes	The results revealed a low Cronbach’s alpha of 0.58 for the scores across the 7 tasks, indicating suboptimal internal consistency. EFA identified two main factors: the first factor primarily associated with rotatory stability and the second factor linked to deep squat, hurdle step, and inline lunge tasks. These two factors accounted for 47.3% of the total variance, suggesting a lack of unidimensionality in the FMS among elite athletes.
Liaghat et al. [42]	Joint hypermobility and shoulder injuries	Systematic review, meta-analysis	2021	Athletes and military personnel	Athletes with joint hypermobility were found to be significantly more likely to experience shoulder injuries compared to those without joint hypermobility, with an odds ratio of 3.25.
Lietz et al. [43]	Risk factors of musculoskeletal diseases and pain among dental professionals	Systematic review, meta-analysis	2018	Dental professionals	The prevalence rates of musculoskeletal disorders ranged from 10.8% to 97.9%. The most affected body region was the neck, the lower back, shoulder, and upper back. Identified potential occupational risk factors included awkward working postures, a high number of treated patients, involvement in administrative work, exposure to vibration, and repetitive tasks.
Lisman et al. [44]	Sleep and musculoskeletal injuries in military personnel	Systematic review	2022	Military personnel	Poor sleep quality or inadequate duration was consistently linked with heightened injury rates across the included studies.
Macedo et al. [45]	Occupational loading and spine degeneration	Systematic review, meta-analysis	2019	Occupational workers	The study suggests moderate-grade evidence linking occupational loading to disc degeneration based on signal intensity, while low-grade evidence is found for disc height and disc bulging, albeit with inconsistent results across spinal levels.
Malek et al. [46]	Beighton score for generalized joint laxity	Theoretical framework	2021	NA	The review concludes that clinical practice needs a shift, advocating against relying solely on the BS to distinguish between localized and generalized hypermobility (GJH) or to rule out GJH altogether. Instead, it emphasizes the importance of clinician judgment and a comprehensive assessment aligned with GJH’s full definition.
Matzkin & Garvey [47]	Sex differences in injuries	Review	2019	NA	Female athletes exhibit higher susceptibility to anterior cruciate ligament injury and patellofemoral pain syndrome compared to their male counterparts. Moreover, treatment outcomes also vary significantly between males and females, such as the higher risk of recurrent shoulder instability among males.
Moore et al. [48]	Predicting injury with FMS	Systematic review, meta-analysis	2019	Athlete populations	The FMS showed higher specificity (85.7%) than sensitivity (24.7%), with a positive predictive value of 42.8% and a negative predictive value of 72.5%. The AUC was 0.587, with a likelihood ratio positive of 1.7 and likelihood ratio negative of 0.87. The effect size calculated from the meta-analysis was 0.68.
Moran et al. [49]	Predicting injury with FMS	Systematic review, meta-analysis	2017	Athletes, military, firefighters, police	In male military personnel, there was “strong” evidence of a “small” association between a low FMS composite score (≤14/21) and subsequent injury (pooled risk ratio = 1.47). However, there was “moderate” evidence discouraging the use of FMS composite scores for injury prediction in football (soccer).
Nambiema et al. [50]	Occupational factors and upper body injuries	Prospective cohort	2020	Occupational workers	Age ≥ 45 years was a significant contributor to UEMSD incidence. Female gender contributed to 12% of UEMSD cases. High physical exertion, working with arms above shoulder level, and low social support were associated with UEMSD risk, consistent with previous epidemiological findings. The study’s population attributable fractions highlighted that nearly 30% of UEMSD cases could be avoided by reducing physical exertion below a certain threshold on the RPE Borg scale.
O’Connor et al. [51]	Predicting injuries with FMS	Prospective cohort	2011	Marine officer candidates	An FMS score of ≤14 predicted any injury with a sensitivity of 0.45 and specificity of 0.71, and serious injury with a sensitivity of 0.12 and specificity of 0.94. Additionally, 79.8% of those with scores ≤ 14 had fitness scores < 280, while only 6.6% with fitness scores ≥ 280 had scores ≤ 14.
Pacey et al. [52]	Generalized joint hypermobility and risk of lower limb joint injury	Systematic review, meta-analysis	2010	Athletes	GJH significantly increases the risk of knee injuries but not ankle injuries during contact sports. The biomechanics of ankle and knee joints, with the knee relying more on passive restraints, explain this difference in injury risk between the two joints.
Girard et al. [53]	FMS and athletic performance	Systematic review	2016	Athletes	The researchers found inconsistent evidence linking FMS scores to performance metrics, suggesting limited predictive value. The study highlights the need for further research to clarify FMS’s role in assessing athletic capability.
Pfeifer et al. [54]	Predicting injuries with FMS	Prospective cohort	2019	Youth athletes	Females scored significantly higher than males in the mean FMS™ composite score (females—14.40, males—12.62) and on individual measures including hurdle step (females—1.91, males: 1.65), shoulder mobility (females—2.68, males—2.02), active straight leg raise (females—2.32, males—1.87), and rotary stability (females—1.91, males—1.65). FMS composite scores of <14 and <15 significantly increased the odds of injury (OR = 2.955), but no significant association was found when adjusting for sport.
Pfeifer et al. [55]	Risk factors for ACL injury	Systematic review	2018	NCAA athletes	Extrinsic factors like weather conditions and playing surfaces were linked to injury risks for both sexes. Intrinsic risk factors mainly focused on non-modifiable anatomic traits, with some suggesting the potential for injury mitigation through neuromuscular training programs. Conflicting evidence exists regarding the impacts of certain morphometric characteristics on ACL injury risk, warranting further investigation. Modifiable neuromuscular and biomechanical risk factors like core stability and knee alignment were highlighted, indicating potential avenues for preventative training. Non-modifiable factors such as menstrual cycle phase and genetic predisposition were also noted.
Plisky et al. [56]	Predicting injuries with SEBT	Prospective cohort	2006	Basketball players	The study found high reliability in the SEBT components, ranging from 0.82 to 0.87 (ICC), and excellent reliability of 0.99 for measuring limb length. Logistic regression analysis revealed that players with a significant difference of more than 4 cm in anterior reach distance between the right and left sides were 2.5 times more likely to experience lower extremity injuries. Additionally, female players with a composite reach distance less than 94.0% of their limb length had a 6.5 times higher likelihood of sustaining lower extremity injuries.
Plisky et al. [56]	Validity and reliability of Y-Balance test lower quarter	Systematic review, meta-analysis	2021	Athletes	Intra-rater reliability ranged from 0.85 to 0.91 across the studies. Sex differences were observed in posteromedial and posterolateral directions but not in the anterior reach direction. Differences in composite scores were noted between sports like soccer, basketball, and baseball. Due to the heterogeneity of injury prediction studies, a meta-analysis was not feasible.
Rhon et al. [57]	Musculoskeletal injury risk in military service members	Systematic review, meta-analysis	2022	Military personnel	Occupational activities like kneeling and high physical activity levels, alongside comorbidities such as depression, may influence knee OA risk. Certain socio-economic attributes might also have protective effects.
Rinaldi et al. [58]	Strength deficits in dynamic knee valgus	Review	2022	NA	This study highlights the critical role of gluteal muscles in maintaining proper knee position during various exercises like walking, running, jumping, and landing, emphasizing their potential in preventing knee injuries.
Ruan et al. [59]	Sleep quality and injuries	Prospective cohort	2021	Military personnel	There was a significantly lower incidence of musculoskeletal trauma injury (MTI) in group 1 (good sleep) (23.6%, 48/203) compared to group 2 (poor sleep) (41.7%, 150/360). Logistic regression indicated that the odds of MTI were 2.307 times higher in group 2 than group 1 (OR = 2.307). Adjusting for age, ethnicity, education, and income (OR = 2.285) or adding body mass index (OR = 2.377) yielded similar results. Group 2 also had approximately 2.1 times higher odds of soft tissue injury than group 1.
Silverwood et al. [60]	Risk factors for knee osteoarthritis in older adults	Systematic review, meta-analysis	2015	Older adults	Known factors like increased BMI, previous knee injury, age, female sex, and hand OA continue to show significance. Smoking remained unassociated with knee OA onset, contrasting other identified risk factors. Weight loss remains crucial, attributing a notable percentage of knee pain cases to overweight or obesity.
Singh et al. [61]	Beighton score cut-offs	Cross-sectional	2017	Australian population	The study found that females and non-Caucasians consistently had higher Beighton scores throughout life, with statistical significance. Using a binary logistic regression model, the Beighton scoring system showed a sensitivity of 0.8% and a specificity of 99.3%, with a cut-off of ≥4. However, this cut-off of ≥4 was deemed suitable only for females aged 40–59 years and males aged 8–39 years, suggesting age and sex-specific considerations for interpreting Beighton scores accurately.
Smith et al. [62]	YBT and injury	Prospective cohort	2015	Division I athletes	The ROC curves identified an asymmetry greater than 4 cm as the optimal cut point for predicting injury, with a sensitivity of 59% and specificity of 72%. Specifically, only anterior asymmetry showed a significant association with noncontact injury, with an odds ratio of 2.33.
Stern et al. [63]	Non-linear nature of injury prediction	Theoretical framework	2020	NA	The study highlights that injuries result from a combination of factors that interact in non-linear ways, making straightforward prediction challenging. The findings suggest that adopting non-linear models could improve the accuracy of injury predictions and inform more effective prevention strategies.
Stroud et al. [64]	Obesity and mechanisms of injury	Systematic review, meta-analysis	2018	Injury patients	The results indicate a higher morbidity rate in obese patients with surgical BBMI compared to non-obese patients, although mortality rates were similar between the two groups, contrary to the obesity paradox. Similar findings were reported in previous studies, emphasizing obesity as a risk factor for post-injury complications rather than mortality.
Svensson et al. [65]	Performance tests and injury risk	Prospective cohort	2018	Athletes	Despite analyzing 86 muscle injuries during the study, no significant correlation was found between the outcomes of physical performance tests and the risk of lower extremity muscle injuries.
Teyhen et al. [66]	Predicting injuries with FMS	Theoretical framework	2014	NA	The study emphasizes the crucial role of preventing musculoskeletal injuries in civilian and military populations, aiming to enhance physical performance and reduce healthcare costs. While existing tools for injury prediction and return-to-activity readiness show promise, there is a need for further research to enhance their sensitivity, specificity, and overall outcomes.
Teyhen et al. [67]	Risk factors for injury	Prospective cohort	2015	US Army Rangers	Risk factors for increased injury include smoking, prior surgery, recurrent musculoskeletal injuries, limited-duty days for musculoskeletal injury, asymmetrical ankle dorsiflexion, pain during FMS clearing tests, and decreased performance in the 2-mile run and 2-min sit-up test. Presenting with one or fewer predictors had a sensitivity of 0.90, while having three or more predictors showed a specificity of 0.98. The multivariable logistic regression model produced an odds ratio of 4.3, a relative risk of 1.9, and an AUC of 0.64.
Teyhen et al. [68]	Risk factors for injury	Prospective cohort	2020	US Army soldiers	A logistic regression model with 11 key variables was significant, with an adjusted R^2^ of 0.21, an odds ratio of 5.7, a relative risk of 2.5, and an area under the curve of 0.73. Sensitivity was 0.89 for individuals with 2 variables, while specificity was 0.94 for those with 7 or more variables.
Ting & Macpherson [69]	Muscle synergies during postural task	Animal study	2005	Cats	Electromyographic data collected from 8–15 hindlimb muscles of cats exposed to various support surface translations revealed that four synergies could explain over 95% of the automatic postural response across all muscles and directions. Each synergy was linked to specific perturbation directions and correlated with a distinct endpoint force vector under the limb, suggesting that postural synergies represent a neural command signal specifying limb endpoint force within active balance control contexts.
Toohey et al. [70]	Association of previous injury and lower limb injury	Systematic review, meta-analysis	2017	Athlete populations	Previous ACL injury increased the risk of subsequent hamstring injury significantly. Having experienced a lower limb muscular injury in the past was linked to an increased risk of sustaining another lower limb muscular injury but at a different site.
Uehli et al. [71]	Sleep problems and work injuries	Systematic review, meta-analysis	2014	Occupational workers	The systematic review established a link between sleep issues and occupational injuries, offering a quantified assessment of this association for the first time.
Verschueren et al. [72]	Acute fatigue and injury risk	Systematic review	2020	Athletes, active adults	The study finds that acute fatigue negatively impacts specific injury risk factors related to lateral ankle sprain, patellofemoral pain syndrome, and hamstring injuries, but there are limited data regarding iliotibial band syndrome, non-contact ACL injuries, and generic lower extremity injury risk profiles.
Wang et al. [73]	Predicting injuries with FMS	Prospective cohort	2017	Division I college athletes	Athletes with an obese BMI had lower FMS composite scores (12.9) compared to those with normal (14.0) and overweight (14.6) BMI. An FMS score ≤ 11 was linked to earlier injury and higher injury rates compared to scores > 11. Independent predictors of injury included female sex, sport type, higher BMI, and precollegiate surgery. The optimal FMS score cut-off point for injury screening was 13 (sensitivity 48.1%, specificity 62.4%, PPV 50.7%, NPV 60.0%). Lowering the cut-off point to 11 reduced sensitivity to 17.4%, increased specificity to 91.7%, raised PPV to 62.7%, and lowered NPV to 58.1%. The AUC was 0.58.
Welsh et al. [74]	Regional interdependence approach to rehab	Case report	2023	Dancer	The report highlights the importance of addressing hip muscle performance and control of the lower extremity during weight-bearing activities to mitigate patellofemoral joint malalignment and anterior knee pain. The discussion also touches upon gender-specific differences in neuromuscular characteristics during activities like jumping and landing, highlighting how female athletes may be more prone to ACL injury due to factors like reduced knee flexion, greater knee valgus, and less hamstring activation.

## Data Availability

No new data were created.
